# Supplementary material for: Assessment of perceived patient comfort and ease of bedpan handling by caregivers, a cross-sectional survey
Source: PLoS One. 2024 Jul 11;19(7):e0306773. doi: 10.1371/journal.pone.0306773 (PMC11239000; doi:10.1371/journal.pone.0306773)
Supplement: S1 Appendix — (DOCX) [file pone.0306773.s001.docx]

| Questionary Evaluation of bedpan by caregivers |
| --- |

| This questionary aims to evaluate the feeling and satisfaction of caregivers with bedpan manipulation at XXX University Hospital.  The answer would be anonymous. |
| --- |

| **My profile** |
| --- |

1. **I am :**

□ Female

□ Male

□ I don’t want to answer

1. **My profession is**

□ Nurse

□ Assistant nurse

□ Student

□ Other:…………

1. **My age :**

□ From 18 to 30 □ From 31 to 50 □ More than 50

1. **I use a bedpan since**

□ < 1 year □ From 1 to 3 years □ From 4 to 10 years □ More than 10 years

1. **I use a bedpan for my patients**

□ Several time per day □ Several time per week □ Less than 1 time a week □ Several time per month

| **My appreciation for bedpan handling** |
| --- |

1. **On a scale of 1 to 5, I find the bedpan installation**

| 1------------ | ----------2----------- | ---------- 3 --------- | ---------- 4 --------- | ---------- 5 |
| --- | --- | --- | --- | --- |
| Not at all difficult | Somewhat not difficult | Neither difficult nor not difficult | Somewhat difficult | Very difficult |

1. **On a scale of 1 to 5, I find the withdrawal of the bedpan**

| 1------------ | ----------2----------- | ---------- 3 --------- | ---------- 4 --------- | ---------- 5 |
| --- | --- | --- | --- | --- |
| Not at all difficult | Somewhat not difficult | Neither difficult nor not difficult | Somewhat difficult | Very difficult |

1. **On a scale of 1 to 5, I find the handle**

| 1------------ | ----------2----------- | ---------- 3 --------- | ---------- 4 --------- | ---------- 5 |
| --- | --- | --- | --- | --- |
| Not at all comfortable | Somewhat uncomfortable | Neither comfortable nor uncomfortable | Somewhat comfortable | Very comfortable |

1. **When using it, I observe that the bedpan tips over**

□ Never □ Sometimes □ Often □ Very often

| **Patient well-being** |
| --- |

1. **On average, I remove the bedpan after**

□ 5 minutes or less □ From 6 to 10 minutes □ From 11 to 15 minutes □ more than 15 minutes

1. **By the time I remove the bedpan, the patients have completely eliminated**

| 1------------ | ----------2----------- | ---------- 3 --------- | ---------- 4 |
| --- | --- | --- | --- |
| I do not at all agree. | Somewhat disagree | Somewhat agree | Strongly agree |

1. **In general, if the patient does not eliminate or minor, it is because of**

| Their health condition | | □ Never | □ Sometimes | □ Often | □ Very often |
| --- | --- | --- | --- | --- | --- |
| Lack of time | | □ Never | □ Sometimes | □ Often | □ Very often |
| Physical discomfort | | □ Never | □ Sometimes | □ Often | □ Very often |
|  |  | |  |  |  |

1. **Other causes of the patient's poor elimination?**

| **General** |
| --- |

1. **Do you have any other comments about the bedpan, the handle, and their use?**
2. **Do you have any ideas for improving the bedpan?**

Thank you for your participation!
